# Functional characterization of NADPH-cytochrome P450 reductase from *Bactrocera dorsalis*: Possible involvement in susceptibility to malathion

**DOI:** 10.1038/srep18394

**Published:** 2015-12-18

**Authors:** Yong Huang, Xue-Ping Lu, Luo-Luo Wang, Dong Wei, Zi-Jiao Feng, Qi Zhang, Lin-Fan Xiao, Wei Dou, Jin-Jun Wang

**Affiliations:** 1 Key Laboratory of Entomology and Pest Control Engineering, College of Plant Protection, Southwest University, Chongqing 400716, P. R. China

## Abstract

NADPH cytochrome P450 reductase (CPR) is essential for cytochrome P450 catalysis, which is important in the detoxification and activation of xenobiotics. In this study, two transcripts of *Bactrocera dorsalis CPR* (*BdCPR*) were cloned, and the deduced amino-acid sequence had an N-terminus membrane anchor for BdCPR-X1 and three conserved binding domains (FMN, FAD, and NADP), as well as an FAD binding motif and catalytic residues for both BdCPR-X1 and BdCPR-X2. *BdCPR-X1* was detected to have the high expression levels in adults and in Malpighian tubules, fat bodies, and midguts of adults, but *BdCPR-X2* expressed lowly in *B. dorsalis*. The levels of *BdCPRs* were similar in malathion-resistant strain compared to susceptible strain. However, injecting adults with double-stranded RNA against *BdCPR* significantly reduced the transcript levels of the mRNA, and knockdown of *BdCPR* increased adult susceptibility to malathion. Expressing complete *BdCPR-X1* cDNA in Sf9 cells resulted in high activity determined by cytochrome *c* reduction and these cells had higher viability after exposure to malathion than control. The results suggest that *BdCPR* could affect the susceptibility of *B. dorsalis* to malathion and eukaryotic expression of *BdCPR* would lay a solid foundation for further investigation of P450 in *B. dorsalis*.

The oriental fruit fly, *Bactrocera dorsalis* (Hendel), is one of the most economically important fruit fly pests in East Asia and the Pacific region, where it endangers a wide range of tropical, subtropical, and temperate fruit crops^1^,^2^. The fruits are damaged by puncturing during oviposition and by the developing larvae feeding on the pulp^3^. Insecticides are a principal tool for pest control worldwide^4^, but resistance in the oriental fruit fly has evolved in some areas^5^,^6^. A thorough understanding of insecticide metabolism and resistance mechanisms is needed for effective pest control. Many studies have suggested that cytochrome P450 enzyme systems are involved in pesticide resistance in many insect species^7^,^8^,^9^,^10^. The combination of piperonyl butoxide (PBO) synergism and P450 monoxygenase activity data implicated the function of P450 enzymes in the insecticide resistance of *B. dorsalis*^6^, and several P450 genes were significantly induced after exposure to insecticides in our previous study^11^.

Cytochrome P450 monooxygenases constitute one of the largest superfamilies of enzymes that act as the terminal oxidase in monooxygenase systems^12^. P450s play key roles in the detoxification and activation of xenobiotics such as drugs, pesticides, plant toxins, and mutagens^13^. Most eukaryotic P450s are dependent microsomal enzymes that, coupled with NADPH cytochrome P450 reductase (CPR), constitute the first step(s) in xenobiotic detoxification^14^,^15^. CPR belongs to a family of flavoproteins that use the flavin cofactors FAD and FMN. It transfers the hydride ion of NADPH to FAD, and then FAD transfers electrons to FMN, which in turn reduces the P450 enzyme heme center to activate molecular oxygen^16^. Thus, the transfer of electrons from NADPH to the P450 heme center by CPR is essential for P450-catalysed metabolism. Moreover, CPR can transfer electrons to other oxidases as well^17^,^18^, and was reported to function in astaxanthin biosynthesis in *Xanthophyllomeces dendrorhous*^19^.

Insect CPR has been considered a vital part of P450-mediated insecticide resistance in species such as *Anopheles gambiae*^20^ and *Cimex lectularius*^21^ because of its key biological role in P450 enzyme systems. However, there is no report about the sequence and characterization of CPR in *B. dorsalis* and whether inactivation of *BdCPR* could reduce the resistance to insecticides. In the current study, two isoforms of CPR from *B. dorsalis* were cloned and characterized. Developmental, sexual, and spatial expression patterns of *BdCPR-X1 and BdCPR-X2*, and expression levels between susceptible and resistant strains were examined by reverse transcription-qPCR (RT-qPCR). Knockdown of *BdCPR* by RNA interference (RNAi) increased the susceptibility of *B. dorsalis* to malathion, and *BdCPR* was functionally expressed in Sf9 cells with typical CPR activities, affecting the susceptibility of Sf9 cells to malathion. The data collected will elucidate the role of CPR-related pathways in susceptibility of *B. dorsalis* to malathion.

## Results

### cDNA cloning and characterization

**Figure 1:**
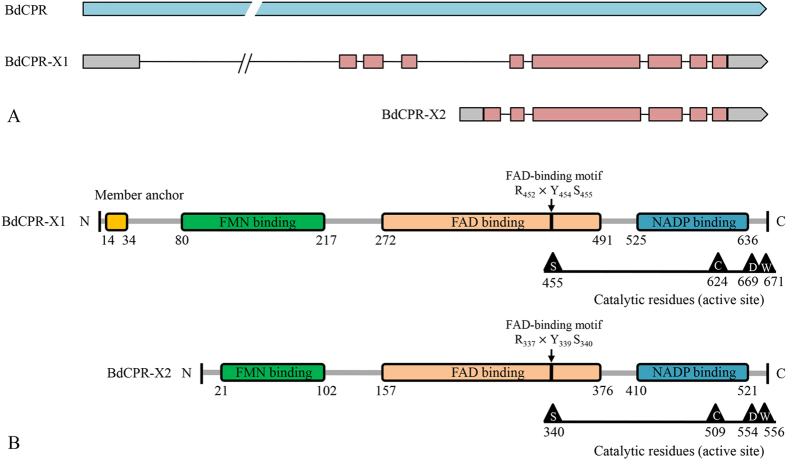
The *Bactrocera dorsalis* CPR (*BdCPR*) gene encodes two isoforms. (**A**) Genome browser view of *BdCPR* gene and its two different isoforms. (**B**) Schematic drawing of BdCPR with membrane anchor (yellow bar), conserved binding domains (flavodoxin, green bar; FAD binding, orange bar; NADP binding, blue bar), FAD binding motif, and catalytic residues.

A cDNA fragment of 680 bp encoding a P450 reductase ortholog was amplified by RT-PCR using degenerate primers and subjected to 5′ and 3′ RACE. A fragment of approximately 1,500 bp was isolated by 3′ RACE, but no fragment was amplified by 5′ RACE. Part of the *BdCPR* cDNA overlapping with the 680 bp fragment was sequenced in the *B. dorsalis* transcriptome^22^,^23^ and used to design the primers CPR5R1 and CPR5R2, and a fragment of ~600 bp was then isolated by 5′ RACE. Full-length *BdCPR* (which was identified as *BdCPR-X1*, GenBank accession number GU325631) was obtained by assembling the four fragments, and the ORF was identified by PCR with a pair of specific primers (Table S1, Supplementary Fig. 1). During the experiments, part of genome and mRNA sequences of *B. dorsalis* was released in NCBI with which RACE primers were designed for the short transcript *BdCPR-X2* (Table S1). As a result, the complete cDNA of *BdCPR-X1* contained an ORF of 2019 bp encoding 672 amino acids, while *BdCPR-X2* cDNA contained an ORF of 1674 bp encoding 557 aa (Fig. 1, Supplementary Figs 2 and 3). The deduced protein of *BdCPR-X1* and *BdCPR-X2* had a calculated molecular weight of 76,221 Da and 63,973 Da, and a theoretical pI of 5.51 and 7.23, respectively.

No signal peptide was observed within the deduced protein sequences of *BdCPR* using SignalP 4.1 Server (Supplementary Fig. 4), but an N-terminal membrane anchor (14E–34W: EPFLGTLDIAILVALIAGATW) was predicted for BdCPR-X1 (Fig. 1, Supplementary Fig. 5). The function domains involved in the binding of cofactors FMA, FAD, and NADPH were identified as primary structures of BdCPR (Fig. 1). Three amino acid residues (Arg 452, Tyr 454, and Ser 455 in BdCPR-X1, and Arg 337, Tyr 339, and Ser 340 in BdCPR-X2) constituted an FAD binding motif, and the BdCPR catalytic residues (active site) were comprised of Ser 455, Cys 624, Asp 669, and Try 701 in BdCPR-X1, and Ser 340, Cys 509, Asp 554, and Try 556 in BdCPR-X2 (Fig. 1). The binding pockets of FAD and NADPH in BdCPR were composed of 13 and 15 amino-acid residues, respectively (Supplementary Fig. 6).

### Phylogenetic relationships and sequence similarities with other CPRs

**Figure 2:**
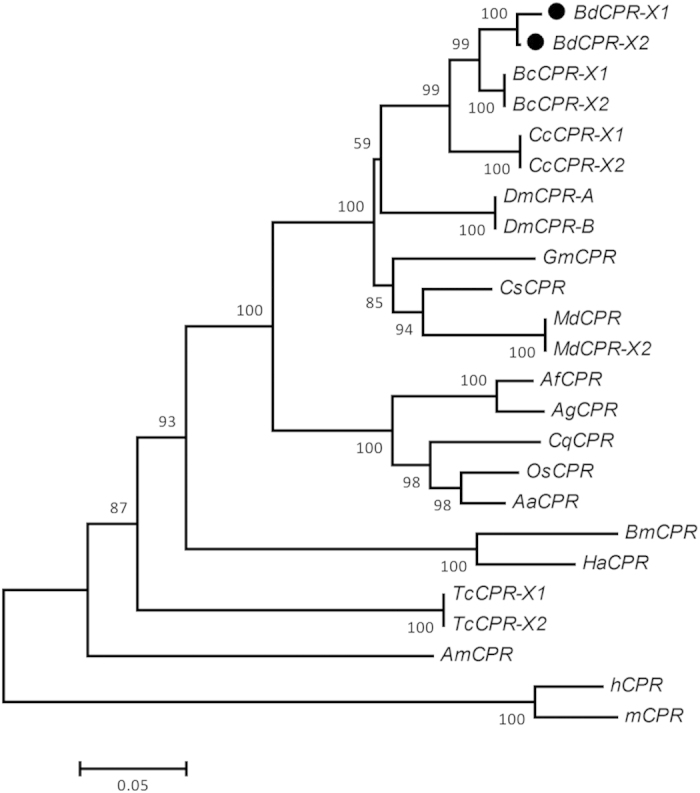
Phylogram of CPRs in insects. The phylogenetic tree was generated by MEGA5 using the neighbor-joining method. CPRs from human and mouse were used as the outgroup. Numbers at nodes are bootstrap percentages (1,000 replicates). Aa, *Aedes aegypti*; Af, *Anopheles funestus*; Ag, *Anopheles gambiae*; Am, *Apis mellifera*; Bc, *Bactrocera cucurbitae*; Bd, *Bactrocera dorsalis*; Bm, *Bombyx mori*; Cc, *Ceratitis capitata*; Cq, *Culex quinquefasciatus*; Cs, *Calliphora stygia*; Dm, *Drosophila melanogaster*; Gm, *Glossina morsitans*; Ha, *Helicoverpa armigera*; h, *Homo sapiens*; Md, *Musca domestica*; m, *Mus musculus*; Os, *Ochlerotatus sollicitans*; Tc, *Tribolium castaneum*.

Phylogenetic analysis was performed on the amino-acid sequences of BdCPR and 20 other CPR proteins from 15 insects, with CPRs of human and mouse as the outgroup, using MEGA 5 with the NJ algorithm. The CPRs from the same taxonomic order generally grouped together, and BdCPRs fell within the Diptera clade. BdCPRs were sister to the CPRs from another *Bactrocera* species, *B. cucurbitae* (BcCPR), and close to *C. capitata* CPRs (CcCPR) (Fig. 2); all of these fruit flies belong to Tephritidae.

The sequences of BdCPR-X1 and BdCPR-X2 showed higher than 90% amino-acid identity with orthologous isoforms from *B. cucurbitae* and *C. capitata* (Table 1). For CPRs from other flies like house fly and fruit fly, they shared more than 80% amino-acid identity with BdCPRs. The sequences of CPR are highly conserved among closely related species. However, relatively low identities were shared with mammals such as *Homo sapiens* (56%).

### Developmental and sexual expression patterns of *BdCPR* mRNA

**Figure 3:**
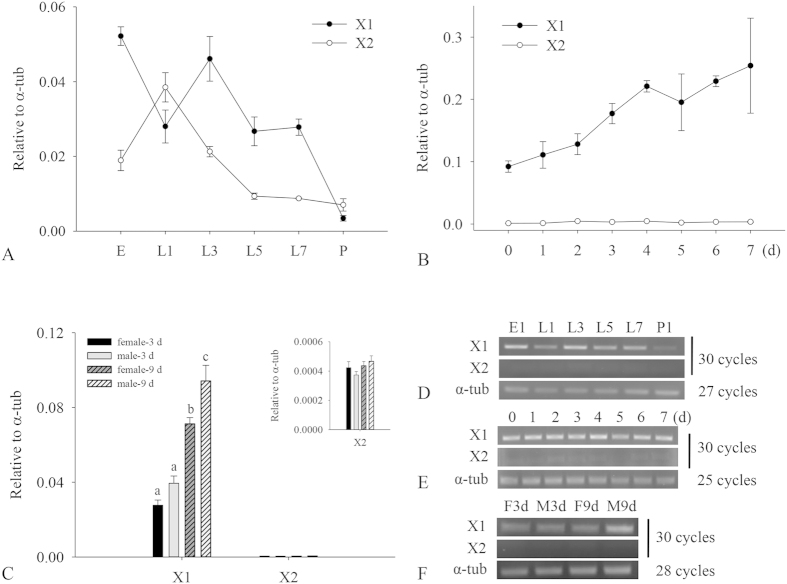
Relative expression levels of *BdCPR* at different developmental stages and between females and males. (**A,D**) The levels of X1 and X2 during development from eggs to pupae. E, egg; L1, 1-d larva; L3, 3-d larva; L5, 5-d larva; L7, 7-d larva. (**B,E**) Their levels in adults at different ages. (**C,F**) Different levels of X1and X2 between 3-d or 9-d old females and males. The data shown are mean ± SE (*n* = 3). Different letters above the error bars indicate statistical differences determined by one-way ANOVA analysis followed by Tukey’s test (*P* < 0.05).

The mRNA levels of *BdCPR* in eggs, larvae, pupae, and adults were quantified using RT-qPCR. *BdCPR-X1* could be detected at all developmental stages and its levels fluctuated during development, declined to a nadir in pupae (Fig. 3A,D). The levels of *BdCPR-X1* increased gradually with the development of adults (Fig. 3B,E), however, relatively low expression levels of *BdCPR-X2* were detected at different stages especially after L5 (Fig. 3A,B,D,E). To know sexual expression patterns of *BdCPR*, 3-d-old and 9-d-old females and males were examined respectively, and the results showed that *BdCPR-X1* was higher in males than in females of the same age, and higher in older females and males (Fig. 3C,F). Differently, the levels of *BdCPR-X2* were low and similar in females and males at those two ages (Fig. 3C,F).

### *BdCPR* mRNA levels in different tissues from females and males

**Figure 4:**
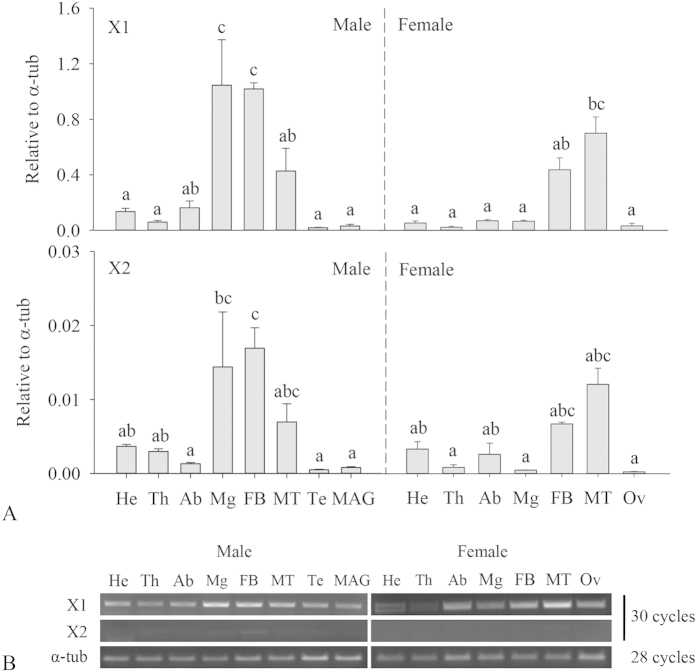
Spatial distribution of *BdCPR* in females and males. The data shown are mean ± SE (*n* = 3). Different letters above the error bars indicate statistical differences determined by one-way ANOVA analysis followed by Tukey’s test (*P* < 0.05). He, head; Th, thorax; Ab, abdomen; Mg, midgut; FB, fat body; MT, Malpighian tubule; Te, testis; MAG, male accessory gland; Ov, ovary.

The *BdCPR* mRNA levels in three body parts including heads, thoraxes, and abdomens, as well as in tissues including midguts, fat bodies, Malpighian tubules, and reproductive organs were compared in this study. *BdCPR-X1* expressed in all tested tissues but with different levels. High expressions were detected in fat bodies and Malpighian tubules in both female and male (Fig. 4A,B). Interestingly, it highly expressed in male midguts but not in female midguts. Reproductive organs in males (testis and male accessory glands) and females (ovaries) had low levels of *BdCPR-X1*. *BdCPR-X2* had similar expression patterns in these tissues with *BdCPR-X1* though the levels of *BdCPR-X2* were low (Fig. 4A,B).

### Transcription profiling in malathion-resistant and -susceptible strains

**Figure 5:**
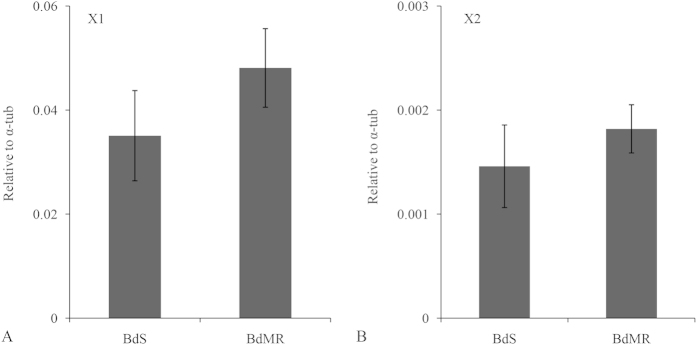
*BdCPR* levels in malathion resistant and susceptible strains. The levels of *BdCPR-X1*(**A**) and *BdCPR-X2* (**B**) in adults from BdS and BdMR strains.

Transcriptional changes of *BdCPR* in malathion resistant and susceptible strains were determined by RT-qPCR. *BdCPR-X1* expression was similar in resistant and susceptible adults, without significant difference (Fig. 5A). *BdCPR-X2* expressed much lowly in both strains and no significant difference could be detected between resistant and susceptible strains (Fig. 5B). The expression of *BdCPR-X1* and *BdCPR-X2* were compared to reference gene ribosomal protein S3 (RPS3) instead of α-tub and showed similar results to α-tub (Supplementary Fig. 7).

### Knockdown of *BdCPR* by RNAi and susceptibility to malathion

**Figure 6:**
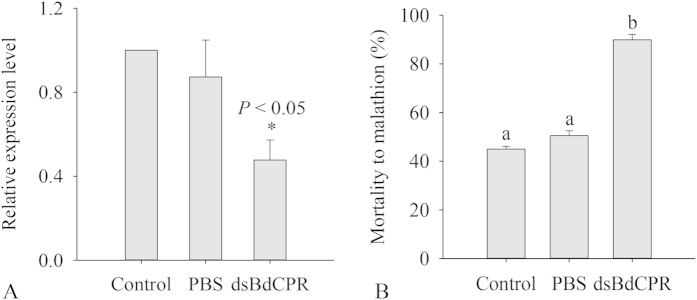
Knockdown of *BdCPR* using RNAi. (**A**) *BdCPR* mRNA levels in *Bactrocera dorsalis* after dsRNA injection. (**B**) Mortality of *B. dorsalis* adults exposed to malathion. Control, non-injected adults; PBS, adults injected with PBS; *dsBdCPR*, adults injected with *dsBdCPR*. The data shown are mean ± SE (*n* = 3). Differences between injected adults and the control were analyzed by Student’s *t*-test. Different letters above the error bars indicate statistical differences determined by one-way ANOVA analysis followed by Tukey’s test (*P* < 0.05).

The dsRNA was designed and amplified based on the common regions in these two *BdCPR* transcripts. Preliminary results showed that the injection of *BdCPR* dsRNA caused low RNAi efficiency at 48 h but high efficiency at 72 h (data not shown). RT-qPCR analysis showed that *BdCPR* mRNA levels decreased dramatically by 52% at 72 h, while injection of PBS did not affect *BdCPR* expression (Fig. 6A). The susceptibility to malathion, indicated by mortality, of adults injected with *dsBdCPR* was significantly higher than in the control and in adults injected with PBS (*P* < 0.05). Mortality of adults injected with *dsBdCPR* reached 89.9%, while it was 45.0% and 50.5% in the control and in adults injected with PBS, respectively (Fig. 6B).

### Heterologous expression of *BdCPR* and activity testing

**Figure 7:**
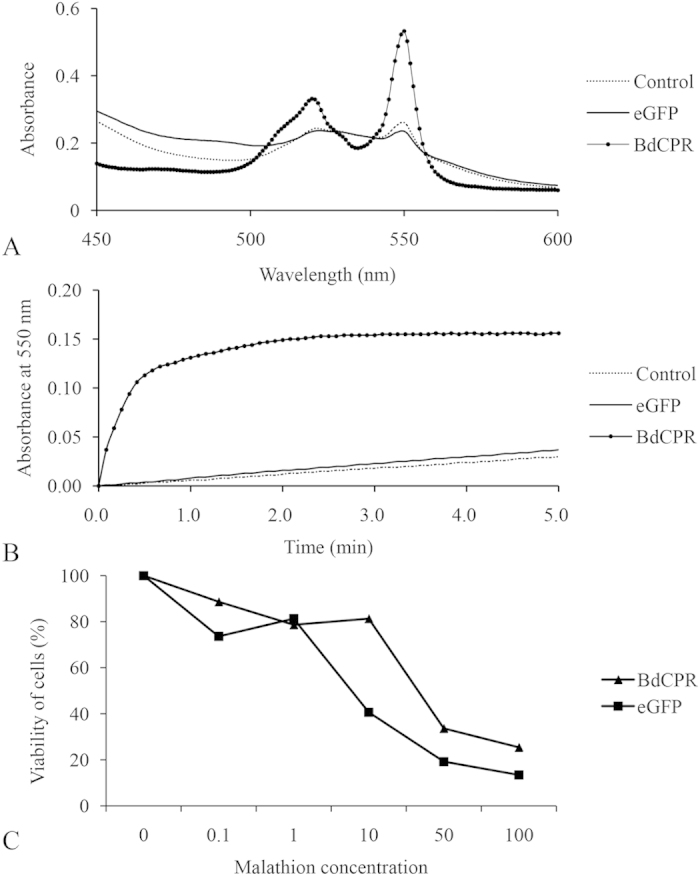
Activity of BdCPR expressed in Sf9 cells measured by cytochrome *c* reduction and viability of cells to malathion. (**A**) Absorption spectra from 450 to 600 nm. (**B**) Time course of cytochrome *c* reduction monitored at 550 nm. (**C**) Viability of eGFP- and BdCPR-expressing cells on incubation with malathion. Control, cell-free extracts of Sf9 cells; eGFP, cell-free extracts of Sf9 cells expressing eGFP; BdCPR, cell-free extracts of Sf9 cells expressing *BdCPR*.

To detect a functional protein encoded by *BdCPR* which could be applied into P450 enzyme system study, the complete ORF of *BdCPR-X1* was cloned into the pFastBac HT A vector with an N-terminus membrane anchor, and the recombinant bacmid DNA was transfected into Sf9 cells and expressed as a 6 × His-tagged fusion protein. Western blot analysis of cell extracts with Anti-His antibody showed that the cells successfully expressed either eGFP or BdCPR (Supplementary Fig. 8). NAPDH-cytochrome P450 reductase activity of the *BdCPR* cDNA-expressed enzyme was determined as described in Methods. The absorbance at 550 nm showed a high level of cytochrome *c* reduction activity with the BdCPR protein product, while an extremely low-level activity with the eGFP enzyme and control cell-free extract (with no transfection) (Fig. 7A). Time course measurements revealed a clear reductase activity in the extracts of Sf9 cells transfected with *BdCPR* recombinant bacmid DNA, but not in the extracts of cells transfected with eGFP recombinant bacmid DNA and in control cells (Fig. 7B). The absorption spectra corresponded to that for cytochrome *c* in the reduced state. These results confirm the activity of the protein product encoded by the isolated *BdCPR*. MTT assay results revealed that the cells expressed with BdCPR had slightly higher viability against different concentrations of malathion than the eGFP-expressing cells (Fig. 7C).

## Discussion

As an obligatory electron donor in the P450 system, CPR transfers electrons from NADPH to the substrate complex to catalyze the metabolism of various endogenous compounds and xenobiotics^24^. Research on CPR in insects would help to determine the function of CPR and the P450 system in the metabolism of insecticides and other xenobiotics^7^,^21^, and might further the development of new methods to manage insecticide resistance. Based on transcriptome datasets and RACE, we identified and cloned the full-length cDNAs containing the complete ORF of *BdCPR* in the present study. Different from the reported insects with one transcript of *CPR*, two *CPR* transcripts were identified in *B. dorsalis*, namely *BdCPR-X1* and *BdCPR-X2*, which is similar to fruit fly and other flies in Tephritidae.

Structural analysis of BdCPR-X1 identified four functional domains. No signal peptide was predicted at its N-terminus, but a hydrophobic transmembrane region of 21 amino acids that might anchor it to the endoplasmic reticulum membrane was found. The anchor region is essential for CPR function in the P450 catalytic cycle by ensuring proper spatial interactions between CPR and P450s^24^,^25^. CPR and cytochrome P450 monooxygenase were both considered to be N-terminally anchored to the endoplasmic reticulum, with the remainder of the enzymes facing the cytoplasm^26^. BdCPR-X1 also had distinct binding domains for FMN, FAD, and NADP. At its N terminus, the FMN binding domain consisted of FMN1 and FMN2 binding sites, which were conserved among insect CPRs, indicating their critical roles in the interaction with P450s. Individual expression of the FMN and FAD/NADP binding domains of CPR showed that they could fold correctly to bind their respective cofactors, but the FAD/NADP domain did not efficiently reduce P450s^27^. However, the domains could be combined to produce a functional enzyme that transferred electrons to P450s, proving that domain assembly was mandatory for CPR function in the P450 system^27^,^28^. The interface between the FMN and FAD domains plays an important role in controlling the redox potential and electron transfer of CPR^29^. However, no member anchor region and short FMN binding region have been identified in BdCPR-X2 (Fig. 1) which might affect its function in electrons transferring like BdCPR-X1. Furth researches should be developed to ascertain the function of BdCPR-X2 though it expressed lowly in *B. dorsalis*.

Highly-conserved primary structures of CPRs across diverse taxa indicated the important roles of this enzyme throughout evolution^25^. An amino acid homology analysis (Blastp) showed that BdCPR shared high identity with homologs in insects, as high as ~95% with those in *B. cucurbitae*, and other animals. Phylogenetic analysis indicated that BdCPR was most closely related to the CPRs of *B. cucurbitae*, consistent with the fact that their CPRs had the highest identity. Though they are conserved in different species, few reports have tested and compared the expression levels of those different transcripts in insects.

The expression levels of *BdCPR* were examined during development and between females and males. *BdCPR-X1* could be detected at different developmental stages and had higher levels in adults, but *BdCPR-X2* had extremely low levels in adults (Fig. 3), which suggested that *BdCPR-X1* might play an important role in the development of *B. dorsalis* and have a key function in adults. High levels of *CPR* in adults were also reported in *C. lectularius*, in which the levels of *C. lectularius CPR* (*ClCPR*) in female and male adults were significantly higher than in the early stages of both deltamethrin resistant and susceptible populations^21^. However, the *CPR* levels varied during development and among insects. For example, *Nilaparvata lugens CPR* (*NlCPR*) levels fluctuated through the developmental stages and had the highest expression in 1st-instar nymphs^30^. *H. armigera CPR* (*HaCPR*) was detected in all tested developmental stages and occurred at the highest levels in 5th-instar larvae^31^. These differences might result from variability in insect physiology and the environments in which they develop. It is interesting that *BdCPR-X1* had higher levels in males than in females of the same age (Fig. 3C,F). It was different from the levels of *CPR* in other species such as *NlCPR* and *ClCPR* which were similar in susceptible females and males^21^,^30^, but it was similar to *ClCPR* which was higher in males than in females in deltamethrin resistant population^21^.

To better understand the different levels of *BdCPR* between females and males, different tissues from females and males were dissected and quantified using RT-qPCR, and the tissue distribution of a protein is usually related to its function. The midgut, fat body, and Malpighian tubule, were tested because of their important physiological roles in insects, especially in metabolism and detoxification. Be in line with the anticipation, *BdCPR* highly expressed in these tissues except for female midguts, and both transcripts had low levels in male and female reproductive system. Consistent with the levels of *BdCPR* were higher in males than in females, the same pattern could be detected in some tissues like midguts between females and males of *B. dorsalis*. The different levels between female and male tissues were also identified in *Chilo suppressalis*, in which *CsCPR* was higher in midguts from males than females^32^. Different from these tissues, *CPR* in *D. melanogaster was* highly expressed in antennae, indicating that its P450s functioned in odorant clearance^33^. *MbCPR* in *Mamestra brassicae* olfactory sensilla reinforced the potential importance of cytochrome metabolism in insect antennae^34^. *AgCPR* was mainly localized in antennae, midgut epithelia, and oenocytes, considered being a major site of heme biosynthesis in insects^20^. Similar to our results, *ClCPR* was broadly present in the head and abdomen^21^, *HaCPR* had high transcript levels in midguts^31^, and *NlCPR* was present at high levels in the abdomen^30^, all potentially implying the roles of CPR and P450s in xenobiotic metabolism and/or endogenous compound biosynthesis.

Owing to its possibility in metabolism and resistance to insecticides, a number of studies have examined the levels of *CPR* in insecticide resistant strains. For instance, the levels of *HaCPR* were significantly higher in midguts from fenvalerate resistant strain than susceptible strain^31^. The transcripts of *PxCPR* in *Plutella xylostella* were significantly more abundant in beta-cypermethrin resistant strain^35^. Such many reports indicated that the levels of *CPR* in insects responded significantly to pyrethroid insecticides which could be metabolized by P450 enzyme system. However, few researches have illustrated the responses of *CPR* to other classes of insecticides. Previous studies showed that *B. dorsalis* has been becoming resistant to malathion which could be mainly metabolized by esterase in the field but P450 enzyme activities increased in malathion-resistant strain^36^. Here, the levels of *BdCPR* between malathion-resistant and -susceptible strains were compared to ascertain the reactions of CPR and even P450 enzyme system to insecticides like malathion. Interestingly, *BdCPR* had similar transcript levels in malathion-resistant strain (BdMR) and malathion-susceptible strain (BdS), which indicated the unnecessary role of BdCPR or even P450 enzyme system in the resistance to malathion in *B. dorsalis*. Thereafter, to identify if the levels of CPR will not affect the susceptibility of *B. dorsalis* to malathion *in vivo*, RNAi was tested in this species.

In insects, *in vivo* inactivation of CPR by inhibitors or the knockdown of target genes by RNAi could enhance insect susceptibility to insecticides. In the mosquito *A. gambiae*, *AgCPR* was highly expressed in oenocytes, and its knockdown increased susceptibility to permethrin^20^. RNAi-mediated knockdown of both *CYP6B7* and *HaCPR* resulted in greater larval susceptibility to fenvalerate than did *CYP6B7* knockdown alone or control treatment^37^. Moreover, silencing *NlCPR* by RNAi significantly increased the susceptibility of *N. lugens* nymphs to beta-cypermethrin and imidacloprid, and the susceptibility of *C. lectularius* to deltamethrin was significantly enhanced after inactivation of *ClCPR* by RNAi^21^. In our study, knockdown of *BdCPR* significantly decreased its mRNA levels in adults and enhanced the susceptibility of *B. dorsalis* to malathion (Fig. 6). This is the first *in vivo* evidence to show the knockdown of *CPR* in *B. dorsalis*. Unexpectedly, decrease of *BdCPR* levels could enhance the susceptibility of *B. dorsalis* to malathion. Different from the similar expression levels of *BdCPR* in malathion resistant strain, several mechanisms could be analyzed to explore the potential reasons. The P450 enzymes and CPR complex could be a major factor involved in the detoxification of various insecticides, and they might metabolize malathion but changes of *BdCPR* levels could not be detected in the medium malathion resistant strain. Although over-expression of P450s such as CYP6G1 in *D. melanogaster* increased the resistance to various insecticides including malathion^38^ and recombinant CYP6G1 exhibited a high binding activity with malathion^39^, further evidences should be shown that P450 enzymes can metabolize malathion and it also happens in *B. dorsalis*. Nevertheless, cuticular penetration of beta-cypermethrin into the larvae of beta-cypermethrin resistant strain was slower than that of susceptible strain associated with the cuticle thickness and integument structure^40^. Knockdown of *CPR* may reduce hydrocarbon production resulting increased penetration of insecticide molecules and thus increase susceptibility to insecticides^41^, which might result in higher susceptibility to malathion in *B. dorsalis*. Therefore, decrease of CPR could enhance the susceptibility of *B. dorsalis* to malathion, but further studies are needed to identify CPR-related pathways in the susceptibility to malathion and P450 genes associated with the insecticide.

Heterologous expression of the P450 system is an efficient method to determine which specific gene functions in detoxification and metabolism. As part of their central roles in drug/insecticide metabolism and biosynthesis of natural compounds^42^, P450s catalyze the introduction of one atom of molecular oxygen into organic molecules, and eukaryotic P450s obtain electrons from CPRs^43^. Thus, expression of highly-active CPR is a key step in this process, since CPR can be the limiting factor for P450 enzyme activity^44^. Heterologous expression of CPR cDNAs with high activity in the house fly *M. domestica*^45^,^46^, fruit fly *D. melanogaster*^47^, and mosquito *An. minimus*^48^ laid a solid foundation for P450 system studies in insects.

CPRs expressed with/without an N-terminus membrane anchor have different activities. Mosquito CPR was expressed in the membrane fraction as a full-length form and in the cytosolic fraction without the N-terminus membrane anchor in *E. coli*^48^. Both expressed products retained cytochrome *c* reducing activity, but it was approximately two-fold higher in cytosol than in membrane form. In the house fly, expressed CPRs in both the membrane and cytosolic fractions could reduce cytochrome *c*, but the products without an N-terminus membrane anchor did not support cytochrome P450 catalysis^46^. The same result was reported in rat, whose CPR was expressed without an N-terminus hydrophobic region and failed to support the cytochrome P450 reaction^49^. However, eukaryotic expression of yeast CPR lacking a 33-amino-acid membrane anchor showed no difference from native yeast CPR in cytochrome *c* reduction and reconstitution of CYP61-mediated sterol Δ^22^-desaturation^50^. BdCPR in this study was expressed in a membrane-anchored form as full length of *BdCPR-X1* to ensure its activity in P450s catalysis in subsequent analyses. The expression of BdCPR in Sf9 cells could lead cells have slightly higher resistance to malathion, which indicated the activity of the expressed CPR protein and it could be functional in Sf9 cells. To date, many insect P450 genes co-expressed with CPR are known to metabolize natural products and insecticides^14^. Therefore, the present expression of *BdCPR* with high activity using a baculovirus expression system provided a solid foundation for future P450 genes studies in this species.

Taken together, two transcripts of *BdCPR* were cloned from *B. dorsalis*. We characterized the sequence and its expression profiles in detail. *BdCPR-X1* was the major CPR in the development and different tissues of *B. dorsalis*. RNAi-mediated knockdown of *BdCPR* increased the susceptibility of *B. dorsalis* to malathion, and expression of BdCPR with high activity was essential for P450-mediated metabolism in *B. dorsalis*. Further investigations into the mechanistic details of CPR in susceptibility to malathion and other insecticides, as well as its interaction with P450s, are necessary.

## Methods

### Insect cultures

The strain of *B. dorsalis* was collected from Guangzhou, Guangdong Province, China, in 2008. The culture conditions and diet were as described previously^11^. Two strains, BdS and BdMR, were developed in our laboratory. BdS was completely susceptible to malathion, while BdMR was selected to be resistant to malathion, with resistance ratios >20-fold^51^.

### Total RNA isolation and cDNA synthesis

Total RNA used to clone the full length of *BdCPR* cDNA was extracted from whole bodies of adults with TRIzol (Life Technologies, Carlsbad, CA, USA). RNA was extracted from malathion-susceptible (BdS) and -resistant (BdMR) adults, and from different developmental stages. RNA was also extracted from specific organs/tissues of adults using an RNAeasy Micro Kit (Qiagen, Hilden, Germany) according to the manufacturer’s protocol. Eight replicates with individual adults (four females and four males) were collected from BdS and BdMR strains. Triple replicates were used for all the other samples.

RNA was quantified by measuring absorbance at 260 nm with a Nanovue UV-Vis spectrophotometer (GE Healthcare, Fairfield, CT, USA), and the quality was ascertained by the absorbance ratio OD260/280 (1.9–2.1). Genomic DNA contamination was eliminated by digesting with DNase I (Takara, Dalian, China). First-strand cDNA for cloning was synthesized from 1 μg of total RNA with a SMARTer RACE cDNA Amplification Kit (Clontech, Palo Alto, CA, USA) and a Perfect Real Time Kit (Takara) for RT-qPCR following the manuals.

### Cloning of full-length cDNA of *BdCPR*

Two degenerate primers (CPR1 and CPR2, Table S1) designed on the basis of conserved amino acid sequences (YGEDPTDN and HPFPCPT) of known CPRs^34^ were used to carry 35 cycles of PCR with annealing temperature of 45 °C. With the fragment cloned, 5′ and 3′ rapid amplification of cDNA ends (RACE) was performed by nested PCR with two gene-specific primers and two universal primers (UPM and NUP, Table S1). The open reading frame (ORF) of *BdCPR* cDNA was amplified and confirmed by PCR methods using specific primers (Table S1). The PCR was carried out with the following procedure: 95 °C of initial incubation for 5 min; followed by 35 cycles of 95 °C for 30 s, 62–68 °C of 30 s, and 72 °C for 1–2 min; and 72 °C of final extension for 10 min. Following purification by agarose gel electrophoresis and gel extraction, the amplified products were cloned into a pGEM-T Easy Vector (Promega, Madison, WI, USA) and transformed into *Escherichia coli* DH5α (TransGen Biotech, Beijing, China). The transformants were screened with Luria–Bertani (LB) agar plates containing 100 μg mL^−1^ of ampicillin, and positive clones were sequenced (Invitrogen, Shanghai, China).

### Sequence analysis and phylogenetic tree construction

Sequences were edited and aligned with ClustelW2 software (www.ebi.ac.uk/Tools/msa/clustalw2/). We searched for similar sequences using Blastp in the non-redundant protein sequences (nr) database of NCBI (http://www.ncbi.nlm.nih.gov). Compute pI/Mw (http://web.expasy.org/compute_pi/) from the Swiss Institute of Bioinformatics was used to estimate the isoelectric point (pI) and molecular weight of the putative BdCPR protein. The signal peptide and protein subcellular localization were predicted using SignalP 4.1 (http://www.cbs.dtu.dk/services/SignalP/). The secondary structure, binding domains, and catalytic residues were predicted with the PHYRE2 Protein Fold Recognition Server (http://www.sbg.bio.ic.ac.uk/phyre2/html/), Pfam 27.0 (http://pfam.sanger.ac.uk/), and a conserved domain search on the NCBI website (http://www.ncbi.nlm.nih.gov/cdd/), respectively. Transmembrane helices were analyzed with TMHMM Server v. 2.0 (http://www.cbs.dtu.dk/services/TMHMM-2.0/). Hydrophobicity was estimated using Protein Hydrophobicity Plots (http://www.vivo.colostate.edu/molkit/hydropathy/), Hphob/Kyte & Doolittle with ProtScale (http://web.expasy.org/protscale/), and DNAMAN software package (v. 6.0; Lynnon Biosoft, Vaudreuil, Quebec, Canada). The phylogenetic tree was constructed in MEGA 5^52^ using the neighbor-joining (NJ) method. CPRs from human and mouse were used as the outgroup. Branch support was estimated by bootstrap analysis with 1,000 replicates.

### Real-time quantitative PCR analysis of *BdCPR*

The RT-qPCR assay was performed in a StepOnePlus (Applied Biosystems, Foster City, CA, USA) using GoTaq qPCR Master Mix (Promega). Each 20 μL final volume contained 400 ng cDNA templates, 10 μL SYBR Green Supermix, 10 pmol each primer (Table S1), and double-distilled water. The reaction was performed under the following conditions: 95 °C for 2 min and 40 cycles of 95 °C for 15 s and 60 °C for 30 s. A dissociation curve from 60–95 °C was included at the end of each RT-qPCR run to verify the specificity of the amplicon for each primer pair. Two technical replicates were run for each biological replicate.

Relative expression levels were calculated by the comparative CT method^53^. Relative quantities were normalized to the stable reference gene α-tubulin evaluated in our previous study^54^,^55^. Expression levels of *BdCPR* between control and injected flies and between susceptible and resistant strains were compared using Student’s *t*-test (two-tailed paired *t*-test). Differences in expression level among developmental stages and tissues were statistically analyzed using one-way ANOVA. Significant differences were further examined with Tukey’s test at a significance level of 0.05 in SPSS 19.0 for Windows (IBM, Chicago, IL, USA).

### Silencing of *BdCPR* by RNAi

*BdCPR* fragments were amplified by PCR using primers, which located in the common sequences between X1 and X2, containing the T7 RNA polymerase promoter (CPRds1 and CPRds2, Table S1). Products were gel-purified and used to synthesize dsRNA with the TranscriptAid T7 High Yield Transcription Kit (Thermo Scientific, Wilmington, DE, USA). The quality of the dsRNAs was confirmed by electrophoresis on an agarose gel, and the concentration was determined with a Nanovue UV-Vis spectrophotometer (GE Healthcare). The dsRNAs was diluted with phosphate buffer saline (PBS, pH 7.0) to a final concentration of 3 μg/μl. Approximately 1 μg dsRNA of *BdCPR* was injected into abdomen between the first and second abdominal segments of each 2-day-old adult with a Nanoject II Auto-Nanoliter Injector (Drummond Scientific, Broomall, PA, USA). Negative controls were injected with an equivalent volume of PBS. Injected and non-injected flies were reared on the artificial diet under conditions described above. Fifty adults were used for each replicate and the mortality caused by injection was controlled in ten percent (less than 5 flies died). The reduction in *BdCPR* transcription levels was determined using three randomly-collected adults 72 h post injection and the remaining adults were used for bioassay. Three replications were used for each treatment, and the primers used for RT-qPCR and RNAi were from separate regions.

### Bioassays after RNAi

The chemical malathion was used to determine mortality in the RNAi analysis as described above. LD_50_ concentrations of malathion were applied to individual insects in the *dsBdCPR*-injected and PBS-injected (negative control) groups 72 h after injection. A group of non-injected (control) insects were also tested. Mortality was checked 24 h post exposure. Three independent bioassays were performed, and nearly 40 adults were tested in each bioassay. Mortality data were subjected to ANOVA and Tukey’s tests at a significance level of 0.05.

### Heterologous expression of *BdCPR* in Sf9 cells

Full-length ORFs of *BdCPR* and eGFP were amplified with primers containing *BamH I* and *Xho I* (Table S1) using PrimeSTAR Max DNA Polymerase (Takara). PCR products and pFastbac HT A vector (Life Technologies) were digested with *BamH I* and *Xho I* (Thermo Scientific) for 1 h and then PCR products were inserted into vectors with T4 DNA Ligase (Promega). The vector containing the eGFP gene was used to produce a control virus. The recombinant baculovirus DNA was constructed and transfected to Sf9 cells (Life Technologies, Gibco) using the Bac-to-Bac baculovirus expression system (Life Technologies) according to the manufacturer’s instructions. Sf9 cells were suspension cultured under serum-free conditions (SF-900 II SFM, Gibco) at 27 °C. Insect cells grown to a density of 2 × 10^6^ cells mL^−1^ were infected with recombinant baculovirus containing either *BdCPR* or eGFP. Baculovirus-infected cells were harvested 72 h post infection by centrifugation at 2,000 × *g* for 10 min and washed with PBS (100 mM, pH 7.8). Cells were resuspended in one-tenth cell culture volume of cell lysate buffer, sonicated for 1 min on ice, and centrifuged at 10,000 × *g* for 10 min. Supernatants were used immediately or frozen in liquid nitrogen and stored at −80 °C.

### Western blot

Concentrations of proteins were determined with the method of Bradford^56^ using bovine serum albumin as a standard. SDS-PAGE was performed using 37.5 μg of total protein for each sample and 12% TGX Stain-Free polyacrylamide gels (Bio-Rad, Hercules, CA), and Precision Plus Protein^TM^ Unstained Standards (Strep-tagged) (Bio-Rad) were loaded parallelly. Separated proteins were transferred to a PVDF membrane using Trans-Blot® Turbo^TM^ RTA Mini PVDF Transfer Kit (Bio-Rad) as the manual. After blocking, proteins were incubated with His-tag primary antibodies (Beyotime biotechnology, Shanghai, China) at a concentration of 1:1,000 for overnight at 4 °C. Then the incubation was changed to HRP-conjugated secondary antibody (1:1,000) (Beyotime) and Precision Protein^TM^ StrepTactin-HRP conjugate (Bio-Rad), with followed incubation with BeyoECL star (Beyotime). Finally, the blotted membrane was imaged using the ChemiDoc XRS^+^ system (Bio-Rad).

### CPR activity assay and cytotoxicity assay

NADPH cytochrome P450 reductase was measured spectrophotometrically by its NADPH-cytochrome *c* reduction activity at room temperature as described previously^57^,^58^. Briefly, the 200 μL reaction mixture contained 16 L of 0.5 mM of a solution of horse heart cytochrome *c* (in 10 mM of potassium phosphate buffer, pH 7.7), 16 μL of protein sample, 166 μL of 0.3 M potassium phosphate buffer (pH 7.7), and 2 μL of 10 mM NADPH. Then, we recorded A_550_ as a function of time (about 5 min) and the absorbance from 450 nm to 600 nm with a 1.0-nm slit width using the Eon Microplate Spectrophotometer (BioTek, Winooski, VT, USA). The extinction coefficient for reduced cytochrome *c* at 550 nm under the described conditions is 21 mM^−1^ cm^−1^.

The cytotoxic effect of malathion to Sf9 cells was evaluated as the method described in our previous paper^51^. Briefly, 500 μL of infected cells was seeded onto a 24-well plate and incubated for 24 h. 10 μL of malathion at different concentrations was added into each well and incubated for 24 h. Using 220 μL MTT solution (200 μL fresh media plus 20 μL MTT) replaced the old media and incubated for 4 h. Removed MTT solution and added 300 μL dimethyl sulphoxide. The absorbance of formazan products were measured at 900 nm with the Spectrophotometer (BioTek) and cell viability was calculated as the percentage of viable cells relative to cells treated with acetone. Triple replicates were performed for each treatment.

## Additional Information

**How to cite this article**: Huang, Y. *et al.* Functional characterization of NADPH-cytochrome P450 reductase from *Bactrocera dorsalis*: Possible involvement in susceptibility to malathion. *Sci. Rep.* **5**, 18394; doi: 10.1038/srep18394 (2015).

## Supplementary Material

Supplementary Information

## Figures and Tables

**Table 1:** Pairwise identities (percent) of predicted amino acid sequences among different CPRs.

	BcCPR	BdCPR	CcCPR	DmCPR	MdCPR
BcCPR	–	**94**	90	83	80
**BdCPR**	**97**	–	**89**	**82**	**80**
CcCPR	95	**94**	–	82	80
DmCPR	88	**86**	86	–	79
MdCPR	86	**85**	86	84	–
hCPR	56	**56**	55	56	56

Below diagonal: X1 isoform; above diagonal: X2 isoform; one CPR isoform from human and two isoforms from each other species; Bc, *Bactrocera cucurbitae*; Bd, *Bactrocera dorsalis*; Cc, *Ceratitis capitata*; Dm, *Drosophila melanogaster*; Md, *Musca domestica*; h, *Homo sapiens*.
