# Supplementary material for: Functional characterization of NADPH-cytochrome P450 reductase from Bactrocera dorsalis: Possible involvement in susceptibility to malathion
Source: Sci Rep. 2015 Dec 18;5:18394. doi: 10.1038/srep18394 (PMC4683403; doi:10.1038/srep18394)
Supplement: Supplementary Information [file srep18394-s1.pdf]

Supplementary Section

Functional characterization of NADPH-cytochrome P450 reductase from *Bactrocera dorsalis*: Possible involvement in susceptibility to malathion

Yong Huang, Xue-Ping Lu, Luo-Luo Wang, Dong Wei, Zi-Jiao Feng, Qi Zhang, Lin-Fan Xiao, Wei Dou & Jin-Jun Wang*

Key Laboratory of Entomology and Pest Control Engineering, College of Plant Protection, Southwest University, Chongqing 400716, P. R. China

Correspondence: Dr. Jin-Jun Wang, College of Plant Protection, Southwest University, Chongqing 400715, P. R. China. E-mail: jjwang7008@yahoo.com, wangjinjun@swu.edu.cn; Tel: (86)-23-68250255; Fax: (86)-23-68251269

Supplementary figures and figure legends

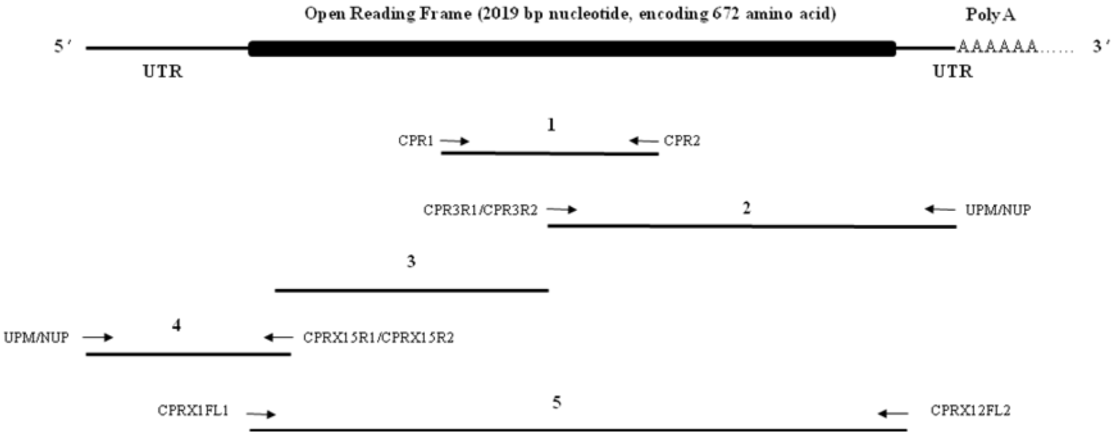

Supplementary Figure 1. Strategy used to clone the full-length *BdCPR-X1*. The top line represents the full-length cDNA. Lines 1, 2, 4, and 5 represent the fragments isolated by PCR and RACE. Line 3 represents the fragment isolated from the transcriptome of *Bactrocera dorsalis*.

```
1       ATGGGGCAGTCATTCATTCGGTTCCATGCTGGAACGACGTACACCTGCTGCTCGTCTGCTCTGCTCTACTCGCTAGTGATCGCATATCGT
91      GCACAAAATCTGTGATTGTATTTGTGAGATTCTGTGTTTATAAGCATTGTTTAGTAATTTTTTCCTGGTTTAATCATTTTTAATGCCAAC
181     AAAAAATATTATTTATTAGTACTTCGAGCAGTTGGTATTAATTATATTGTGTACTTTAATATTTAAAGTGACAATAATATTTTGTATAAA
271     ACTCGAGTAATAATACAACTATATAATAATTTCTCGATAAAACCAGTAAATAAATAAGCATTGAACTGCCTAAACTAGTCTAACGTCTCT
361     CACTCCAAGGAGAGGCATTGTGTGGCTCAAAATCTTGCAGCTTAATCGTTCATTTAAAACGATACAAATTTTAACATAACATCGTACTAT
451     ATATGTATGTATGAACTTGTGCAATAATATTAAGAGAAAATCGTGTAGCAAAAAGTAATTGAAATGTCAGCGGAGAAAATCGAAGAAATC
1                                                                       M  S  A  E  K  I  E  E  I 

541     CCAGTCGGCGATGAACCCTTCTTGGGTACATTGGACATTGCCATACTGGTGGCCTTAATCGCAGGTGCCACATGGTATTTTATGCGTAGT
10       P  V  G  D  E  P  F  L  G  T  L  D  I  A  I  L  V  A  L  I  A  G  A  T  W  Y  F  M  R  S 

631     CGTAAGAAGGAGGAGGAACCCATTCGCTCCTATTCGATACAACCTACAACTGTAAGCACCACCATCTCATCCGACAATTCATTTATTAAA
40       R  K  K  E  E  E  P  I  R  S  Y  S  I  Q  P  T  T  V  S  T  T  I  S  S  D  N  S  F  I  K 

721     AAGCTGAAAGCTTCTGGTCGCAGTTTAGTCGTCTTTTATGGCTCTCAAACGGGTACTGGTGAGGAATTCGCCGGTCGTCTGGCAAAAGAA
70       K  L  K  A  S  G  R  S  L  V  V  F  Y  G  S  Q  T  G  T  G  E  E  F  A  G  R  L  A  K  E 

811     GGCTTACGGTATCGCATGAAGGGTATGGTAGCAGATCCGGAAGAATGTGATATGGAAGAGCTTTTGCAATTGAAAGACATTCCTAATTCT
100      G  L  R  Y  R  M  K  G  M  V  A  D  P  E  E  C  D  M  E  E  L  L  Q  L  K  D  I  P  N  S 

901     CTGGCTGTATTCTGTTTAGCCACATATGGCGAAGGTGATCCAACAGATAATGCAATGGAATTCTTTGAATGGCTGTCAAATGGCGATGCT
130      L  A  V  F  C  L  A  T  Y  G  E  G  D  P  T  D  N  A  M  E  F  F  E  W  L  S  N  G  D  A 

991     GATTTGAATGGATTGAATTATGCGGTTTTCGGCCTTGGCAATAAAACTTATGAGCATTATAACAAAATGGCAATTTATGTGGATCAACGA
160      D  L  N  G  L  N  Y  A  V  F  G  L  G  N  K  T  Y  E  H  Y  N  K  M  A  I  Y  V  D  Q  R 

1081    TTGGAAGAGCTTGGTGCTACACGTGTCTTTGAGCTTGGTCTTGGCGATGACGATGCAAACATTGAGGACGACTTTATTACATGGAAAGAT
190      L  E  E  L  G  A  T  R  V  F  E  L  G  L  G  D  D  D  A  N  I  E  D  D  F  I  T  W  K  D 

1171    CGTTTTTGGCCTGCTGTTTGTGATTTCTTCGGCATTGAGGGAGGCGGAGAGGAAGTGCTTATGCGCCAGTATCGGTTACTGGAGCAACCA
220      R  F  W  P  A  V  C  D  F  F  G  I  E  G  G  G  E  E  V  L  M  R  Q  Y  R  L  L  E  Q  P 

1261    AATGTACAGCCCGATCGCATCTACACCGGCGAAATTGCACGCTTGCACTCTTTGCAAAATCAACGACCTCCATTTGACGCTAAAAATCCA
250      N  V  Q  P  D  R  I  Y  T  G  E  I  A  R  L  H  S  L  Q  N  Q  R  P  P  F  D  A  K  N  P 

1351    TTCCTAGCACCCATTATTGTTAATCGTGAATTACACAAAGGTGGCGACCGTTCATGTATGCATATCGAGCTGGATATTAATGGCTCAAAA
280      F  L  A  P  I  I  V  N  R  E  L  H  K  G  G  D  R  S  C  M  H  I  E  L  D  I  N  G  S  K 

1441    ATGCGTTACGATGCGGGAGATCACGTTGCCATGTATCCTATTAATGATACCGAATTGGTCGAAAAATTGGGAAAACTTTGCAATGCTGAT
310      M  R  Y  D  A  G  D  H  V  A  M  Y  P  I  N  D  T  E  L  V  E  K  L  G  K  L  C  N  A  D 

1531    CTCGATACGGTATTTTCGCTCATTAATACTGATACGGATAGCAGTAAAAAACACCCCTTTCCTTGTCCTACCACTTATCGCACCGCTTTG
340      L  D  T  V  F  S  L  I  N  T  D  T  D  S  S  K  K  H  P  F  P  C  P  T  T  Y  R  T  A  L 

1621    AAACATTATTTGGAAATTACTGCCATACCAAGAACACATATTCTCAAAGAATTGGCGGAGTATTGTACTGACGAGGCCGATAAAGAATTC
370      K  H  Y  L  E  I  T  A  I  P  R  T  H  I  L  K  E  L  A  E  Y  C  T  D  E  A  D  K  E  F 

1711    TTAAGAAGTATGTCTTCAATTTCACCCGAAGGCAAAGAAAAATACCAGAGTTGGATTCAAGATGCTTGTAGAAATATTGTACATATCTTA
400      L  R  S  M  S  S  I  S  P  E  G  K  E  K  Y  Q  S  W  I  Q  D  A  C  R  N  I  V  H  I  L 

1801    GAAGATATCAAGTCCTGCAAACCGCCTATAGATCATATTTGCGAGCTATTGCCTCGTCTGCAGCCGCGCTATTATTCTATATCATCGTCT
430      E  D  I  K  S  C  K  P  P  I  D  H  I  C  E  L  L  P  R  L  Q  P  R  Y  Y  S  I  S  S  S 

1891    TCCAAATTGCATCCAAATCATGTGCATGTAACAGCCGTTTTAGTGCAGTACAAAACACCCACTGGACGCATAAATAACGGCGTAGCCACC
460      S  K  L  H  P  N  H  V  H  V  T  A  V  L  V  Q  Y  K  T  P  T  G  R  I  N  N  G  V  A  T 

1981    ACATACTTGAAAAAGAAACAGCCCGGCGGCGAAGATGTTCGTGTACCGGTATTTATTCGTAAGTCACAATTCAGGCTACCTACAAAACCA
490      T  Y  L  K  K  K  Q  P  G  G  E  D  V  R  V  P  V  F  I  R  K  S  Q  F  R  L  P  T  K  P 

2071    GAAATACCTATTATCATGGTTGGACCGGGCACTGGATTGGCTCCATTCCGTGGTTTCATTCAAGAGCGTCAATATCTCCGCGATGAAGGT
520      E  I  P  I  I  M  V  G  P  G  T  G  L  A  P  F  R  G  F  I  Q  E  R  Q  Y  L  R  D  E  G 

2161    AAAAATGTGGGCGATACCATTTTATATTTCGGCTGTAGAAAGAGAAGTGAAGACTACATTTATGAAGAGGAATTGGAGGAATTTATCAAG
550      K  N  V  G  D  T  I  L  Y  F  G  C  R  K  R  S  E  D  Y  I  Y  E  E  E  L  E  E  F  I  K 

2251    AAGGGCACGTTGACTATGAAAGCAGCTTTCTCTCGTGATCAAAATGAAAAAGTCTATGTTTCACATTTAATTGAAAAGGACGCCGACTTG
580      K  G  T  L  T  M  K  A  A  F  S  R  D  Q  N  E  K  V  Y  V  S  H  L  I  E  K  D  A  D  L 

2341    ATTTGGAATGTGATAGGAGAGAGCAAAGGGCATTTTTACATTTGCGGTGATGCGAAAAACATGGCCGTCGATGTCAGGAATATTTTAATC
610      I  W  N  V  I  G  E  S  K  G  H  F  Y  I  C  G  D  A  K  N  M  A  V  D  V  R  N  I  L  I 

2431    AAGATACTTGTGACGAAGGGTGGCATGAGCGAAGCTGATGCTGTGCAATACCTTAAGAAAATGGAAGCCCAGAAACGCTATTCCGCCGAT
640      K  I  L  V  T  K  G  G  M  S  E  A  D  A  V  Q  Y  L  K  K  M  E  A  Q  K  R  Y  S  A  D 

2521    GTTTGGAGCTAGAAATTTTGACGAAAAACATAATATCTGATCAAGTCTTTAATTCTTGTTAAGTAGATAAATTAGTGTTTTGGCTGTTAT
670      V  W  S  * 

2611    GGCGAAAATGTGTGCATCTTTATATGCATGTGAATGAATACCAGAATAGATAAGTAAAGCAAAGTACATCAACAAAAAAAAAAAAAAAAA
2701    AAAAAAAAAAAAA
```

Supplementary Figure 2. Nucleotide and deduced amino-acid sequences of *BdCPR-X1*.

```
1       GTTTGAATTTTTGTGCAAACCATATATCAGCGCCATCTATATTACGATGTTGAAAATGTTAGGGTTGTCGGCTTTCATATTTCTGAACAG
91      CTGATAACTTACAGCTGTTTTCACCCTACCCTCCATCACGAACTGTCAAAAGTCCAATGTGCTATTGCATAAATCGTTTATTATATTTCC
181     TGTACTAAAGTTTAATAAAAATAATAATAAAAATTAATTTACATTACGCTACAACTTTCATTTGAAGTCATAATTTAATCAACGATAATG
271     AGGAGTTGAACGATAACATGGTGACATAACGTATGAACATGCTGATGAAATTGGGTACATATCGATGGCGCAATCTTGTGCTTTCAAGTG
1                                       M  N  M  L  M  K  L  G  T  Y  R  W  R  N  L  V  L  S  S

361     CATCATTGGAACGACTGAGACACTATCAAAGCGCAGTCGCATTAGATTATTTTTGTAATAATAAAATTAATCTAACATTAAAGACTTTGC
20      A  S  L  E  R  L  R  H  Y  Q  S  A  V  A  L  D  Y  F  C  N  N  K  I  N  L  T  L  K  T  L

451     AAATGAGGGTTTTCGGCCTTGGCAATAAAACTTATGAGCATTATAACAAAATGGCAATTTATGTGGATCAACGATTGGAAGAGCTTGGTG
50      Q  M  R  V  F  G  L  G  N  K  T  Y  E  H  Y  N  K  M  A  I  Y  V  D  Q  R  L  E  E  L  G

541     CTACACGTGTCTTTGAGCTTGGTCTTGGCGATGACGATGCAAACATTGAGGACGACTTTATTACATGGAAAGATCGTTTTTGGCCTGCTG
80      A  T  R  V  F  E  L  G  L  G  D  D  D  A  N  I  E  D  D  F  I  T  W  K  D  R  F  W  P  A

631     TTTGTGATTTCTTCGGCATTGAGGGAGGCGGAGAGGAAGTGCTTATGCGCCAGTATCGGTTACTGGAGCAACCAAATGTACAGCCCGATC
110     V  C  D  F  F  G  I  E  G  G  G  E  E  V  L  M  R  Q  Y  R  L  L  E  Q  P  N  V  Q  P  D

721     GCATCTACACCGGCGAAATTGCACGCTTGCACTCTTTGCAAAATCAACGACCTCCATTTGACGCTAAAAATCCATTCCTAGCACCCATTA
140     R  I  Y  T  G  E  I  A  R  L  H  S  L  Q  N  Q  R  P  P  F  D  A  K  N  P  F  L  A  P  I

811     TTGTTAATCGTGAATTACACAAAGGTGGCGACCGTTCATGTATGCATATCGAGCTGGATATTAATGGCTCAAAAATGCGTTACGATGCGG
170     I  V  N  R  E  L  H  K  G  G  D  R  S  C  M  H  I  E  L  D  I  N  G  S  K  M  R  Y  D  A

901     GAGATCACGTTGCCATGTATCCTATTAATGATACCGAATTGGTCGAAAAATTGGGAAAACTTTGCAATGCTGATCTCGATACGGTATTTT
200     G  D  H  V  A  M  Y  P  I  N  D  T  E  L  V  E  K  L  G  K  L  C  N  A  D  L  D  T  V  F

991     CGCTCATTAATACTGATACGGATAGCAGTAAAAAACACCCCTTTCCTTGTCCTACCACTTATCGCACCGCTTTGAAACATTATTTGGAAA
230     S  L  I  N  T  D  T  D  S  S  K  K  H  P  F  P  C  P  T  T  Y  R  T  A  L  K  H  Y  L  E

1081    TTACTGCCATACCAAGAACACATATTCTCAAAGAATTGGCGGAGTATTGTACTGACGAGGCCGATAAAGAATTCTTAAGAAGTATGTCTT
260     I  T  A  I  P  R  T  H  I  L  K  E  L  A  E  Y  C  T  D  E  A  D  K  E  F  L  R  S  M  S

1171    CAATTTCACCCGAAGGCAAAGAAAAATACCAGAGTTGGATTCAAGATGCTTGTAGAAATATTGTACATATCTTAGAAGATATCAAGTCCT
290     S  I  S  P  E  G  K  E  K  Y  Q  S  W  I  Q  D  A  C  R  N  I  V  H  I  L  E  D  I  K  S

1261    GCAAACCGCCTATAGATCATATTTGCGAGCTATTGCCTCGTCTGCAGCCGCGCTATTATTCTATATCATCGTCTTCCAAATTGCATCCAA
320     C  K  P  P  I  D  H  I  C  E  L  L  P  R  L  Q  P  R  Y  Y  S  I  S  S  S  S  K  L  H  P

1351    ATCATGTGCATGTAACAGCCGTTTTAGTGCAGTACAAAACACCCACTGGACGCATAAATAACGGCGTAGCCACCACATACTTGAAAAAGA
350     N  H  V  H  V  T  A  V  L  V  Q  Y  K  T  P  T  G  R  I  N  N  G  V  A  T  T  Y  L  K  K

1441    AACAGCCCGGCGGCGAAGATGTTCGTGTACCGGTATTTATTCGTAAGTCACAATTCAGGCTACCTACAAAACCAGAAATACCTATTATCA
380     K  Q  P  G  G  E  D  V  R  V  P  V  F  I  R  K  S  Q  F  R  L  P  T  K  P  E  I  P  I  I

1531    TGGTTGGACCGGGCACTGGATTGGCTCCATTCCGTGGTTTCATTCAAGAGCGTCAATATCTCCGCGATGAAGGTAAAAATGTGGGCGATA
410     M  V  G  P  G  T  G  L  A  P  F  R  G  F  I  Q  E  R  Q  Y  L  R  D  E  G  K  N  V  G  D

1621    CCATTTTATATTTCGGCTGTAGAAAGAGAAGTGAAGACTACATTTATGAAGAGGAATTGGAGGAATTTATCAAGAAGGGCACGTTGACTA
440     T  I  L  Y  F  G  C  R  K  R  S  E  D  Y  I  Y  E  E  E  L  E  E  F  I  K  K  G  T  L  T

1711    TGAAAGCAGCTTTCTCTCGTGATCAAAATGAAAAAGTCTATGTTTCACATTTAATTGAAAAGGACGCCGACTTGATTTGGAATGTGATAG
470     M  K  A  A  F  S  R  D  Q  N  E  K  V  Y  V  S  H  L  I  E  K  D  A  D  L  I  W  N  V  I

1801    GAGAGAGCAAAGGGCATTTTTACATTTGCGGTGATGCGAAAAACATGGCCGTCGATGTCAGGAATATTTTAATCAAGATACTTGTGACGA
500     G  E  S  K  G  H  F  Y  I  C  G  D  A  K  N  M  A  V  D  V  R  N  I  L  I  K  I  L  V  T

1891    AGGGTGGCATGAGCGAAGCTGATGCTGTGCAATACCTTAAGAAAATGGAAGCCCAGAAACGCTATTCCGCCGATGTTTGGAGCTAGAAAT
530     K  G  G  M  S  E  A  D  A  V  Q  Y  L  K  K  M  E  A  Q  K  R  Y  S  A  D  V  W  S  *

1981    TTTGACGAAAAACATAATATCTGATCAAGTCTTTAATTCTTGTTAAGTAGATAAATTAGTGTTTTGGCTGTTATGGCGAAAATGTGTGCA
2071    TCTTTATATGCATGTGAATGAATACCAGAATAGATAAGTAAAGCAAAGTACATCAACAAAAAAAAAAAAAAAAAAAAAAAAAAAAAA
```

Supplementary Figure 3. Nucleotide and deduced amino-acid sequences of *BdCPR-X2*.

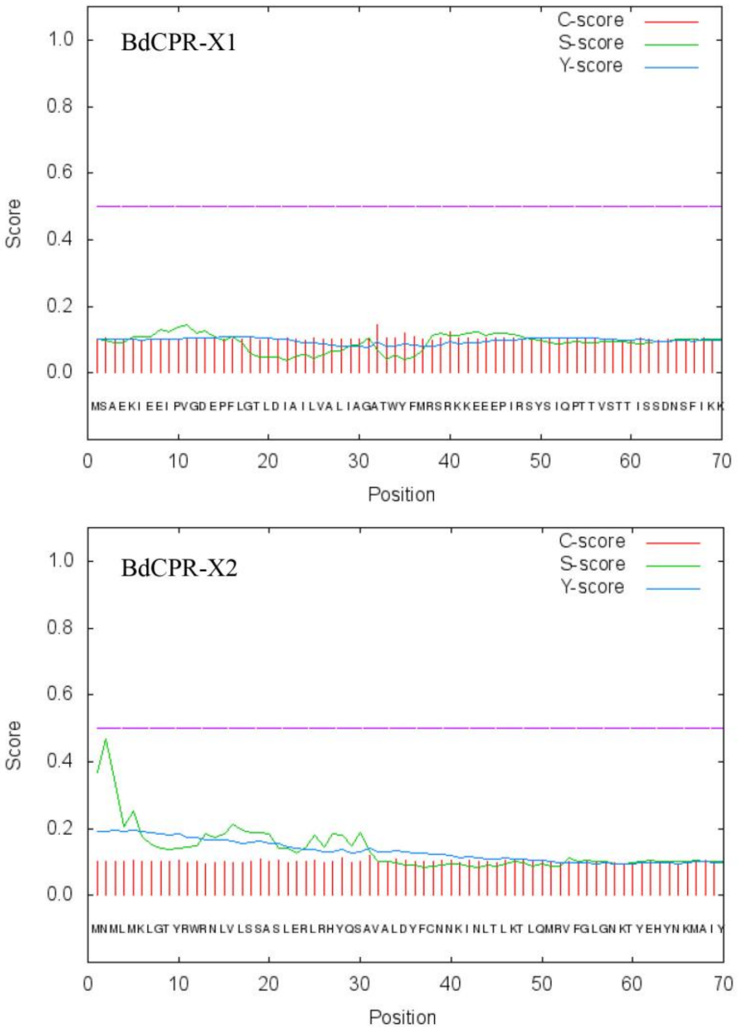

Supplementary Figure 4. SignalP 4.1 prediction of BdCPR.

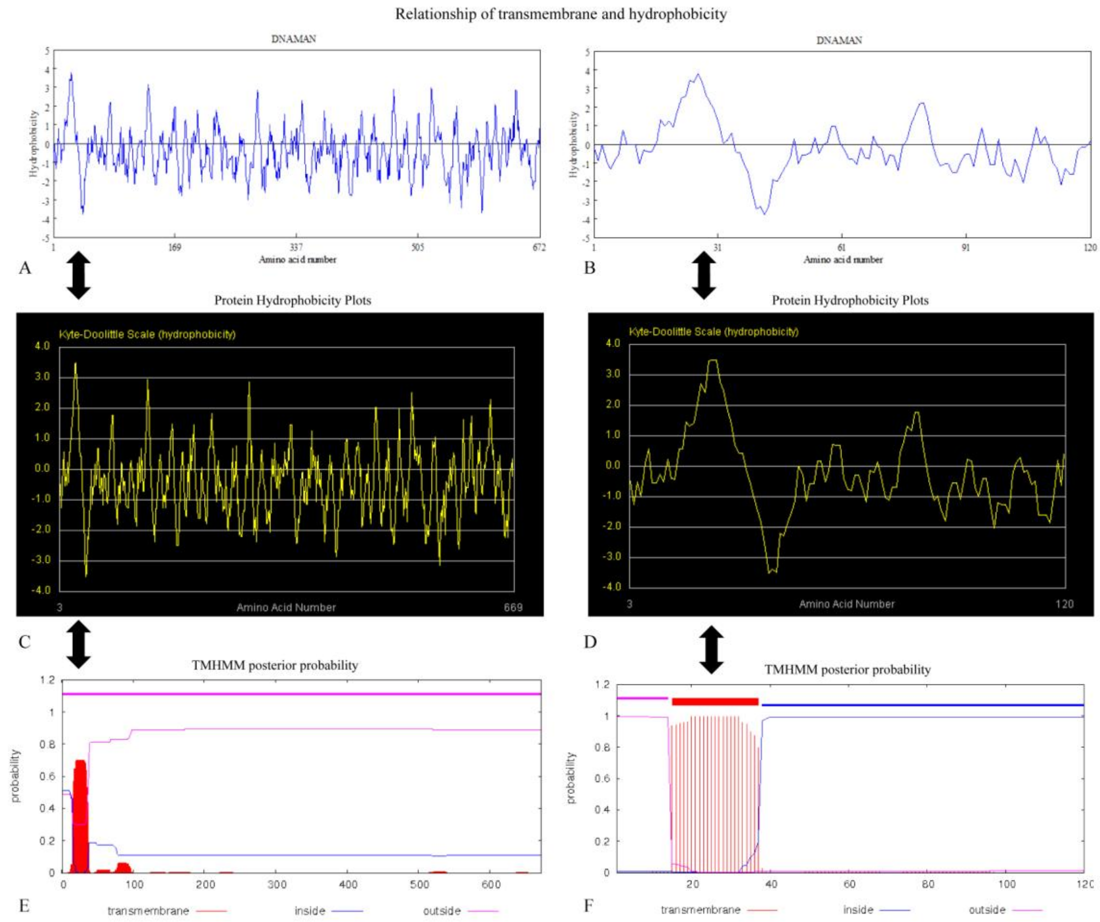

Supplementary Figure 5. Hydrophobicity analysis of BdCPR-X1. A total 672 amino acids and 120 N-terminus amino acids were submitted to DNAMAN (A and B), Protein Hydrophobicity Plots (C and D), and TMHMM Server 2.0 (E and F).

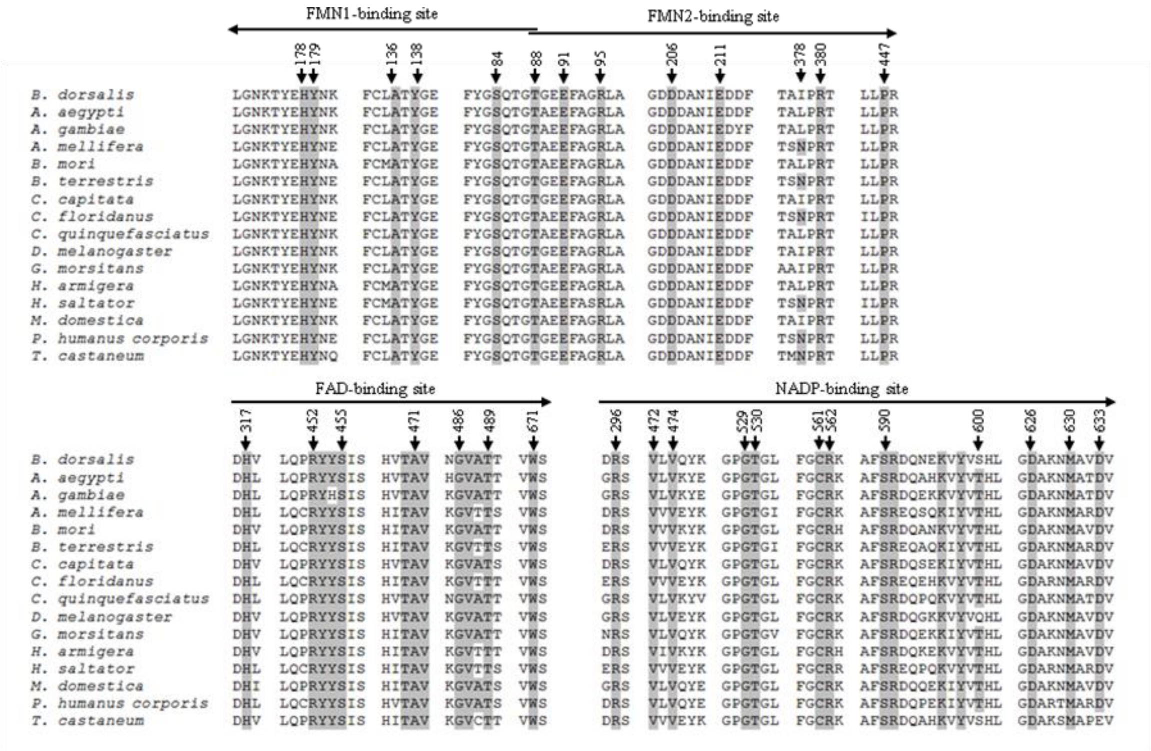

Supplementary Figure 6. Alignment of FMN-, FAD- and NADP-binding sites in insects. Arrows indicate the N-terminus to C-terminus direction. Residues constituting the binding site in each domain are shaded. All insect CPR sequences were retrieved from NCBI.

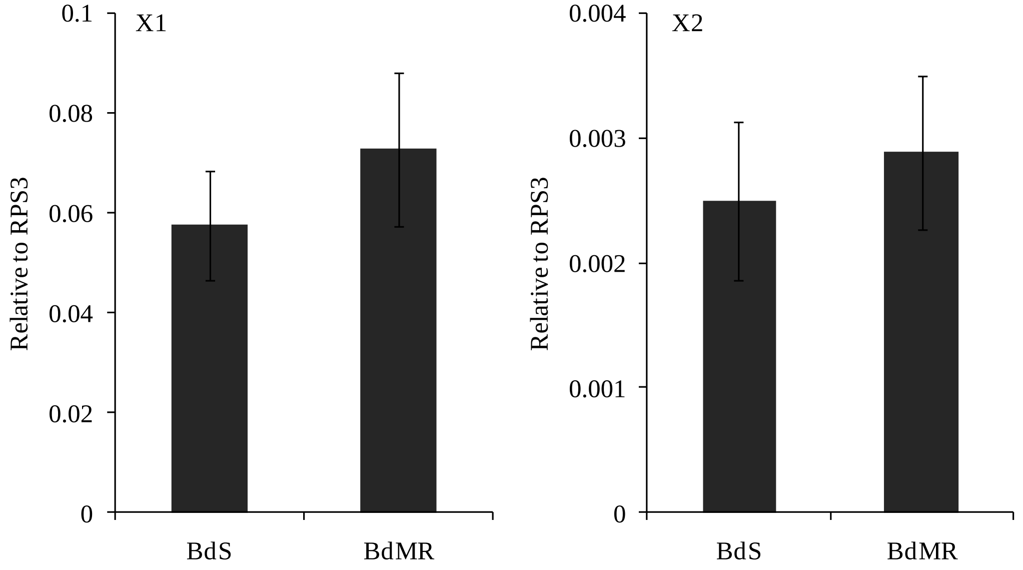

Supplementary Figure 7. Levels of BdCPR-X1 and BdCPR-X2 in malathion resistant (BdMR) and susceptible (BdS) strains.

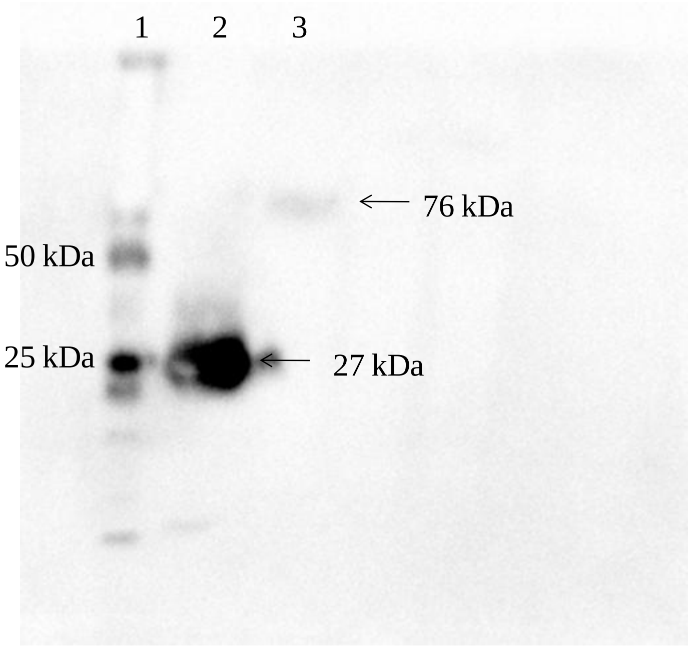

Supplementary Figure 8. Western blot analysis of eGFP- and BdCPR-expressed Sf9 cells. 6×-His tagged eGFP and BdCPR were stained with Anti-His antibody. 1, Marker. 2, Proteins from eGFP-expressed cells. 3, Proteins from BdCPR-expressed cells.

Supplementary tables

Table S1 Primers used in the present study

Gene	Primer name	Primer sequence (5′ →3′)	Function
	CPR1	TAYGGYGARGGMGATCCCAC	Degenerated primers
	CPR2	GTGGGRCARGGGAATGGRTG	—
	CPR3R1	GCCTGCTGTTTGTGATTTCTTCGG	3' RACE
	CPR3R2	GAGGGAGGCGGAGAGGAAGTGC	—
	CPRX15R1	CCTCCTCCTTCTTACGACTACGC	5' RACE
	CPRX15R2	AGGGTTCATCGCCGACTGG	—
	CPRX25R1	GCTTTGATAGTGTCTCAGTCGTTC	—
	CPRX25R2	ACCCAATTTCATCAGCATGTTCATAC	—
	UPM	CTAATACGACTCACTATAGGGCAAGCAGTGGTATCAACGCAGAGT	RACE
	NUP	AAGCAGTGGTATCAACGCAGAGT	RACE
	CPRX1FL1	GTAATTGAAATGTCAGCGG	Cloning full-length cDNA
BdCPR	CPRX2FL1	ATGAACATGCTGATGAAATT	—
	CPRX12FL2	CAGATATTACGTTTTTCGTC	—
	CPRX1Q1	AGCATTGAACTGCCTAAACTAGTC	RT-qPCR
	CPRX1Q2	AGCGAATGGGTTCCTCCTC	—
	CPRX2Q1	CTTACAGCTGTTTTCACCCTACC	—
	CPRX2Q2	GCTTTGATAGTGTCTCAGTCGTTC	—
	CPRds1	TAATACGACTCACTATAGGGGTGCTTATGCGCCAGTATC	RNAi
	CPRds2	TAATACGACTCACTATAGGGTCTTTGCCTTCTGGTGAAA	—
	CPRdsQ1	AGAGCGTCAATATCTCCGCG	RT-qPCR for RNAi
	CPRdsQ2	TCAAGTCGGCGTCCTTTTCA	—
	CPRBES1	CGCGGATCCCATGTCAGCGGAAAAAATCG	Heterologous expression
	CPRBES2	CCGCTCGAGCTAGCTCCAAACATCGGC	—
eGFP	eGFP1	CGCGGATCCCATGGTGAGCAAGGGCGAGGAGCTG	Heterologous expression
	eGFP2	CCGCTCGAGAGGTTCAGGGGGAGGTGTGGGAGG	—
α-tub	α-tub1	CGCATTCATGGTTGATAACG	Reference gene
	α-tub2	GGGCACCAAGTTAGTCTGGA	—
RPS3	RPS3S	TAAGTTGACCGGAGGTTTGG	Reference gene
	RPS3A	TGGATCACCAGAGTGGATCA	—
